# Ultrasound-guided esophageal compression during mask ventilation in small children: a prospective observational study

**DOI:** 10.1186/s12871-022-01803-5

**Published:** 2022-08-15

**Authors:** Eun-Hee Kim, Sung-Ae Cho, Pyoyoon Kang, In-Sun Song, Sang-Hwan Ji, Young-Eun Jang, Ji-Hyun Lee, Jin-Tae Kim, Hee-Soo Kim

**Affiliations:** 1 Department of Anesthesiology and Pain Medicine, Seoul National University Hospital, Seoul National University College of Medicine, 101 Daehakno, Jongnogu Seoul, 03080 Republic of Korea; 2 Department of Anesthesiology and Pain Medicine, Konyang University Hospital, Konyang University College of Medicine, 158 Gwanjeodong-ro, Seo-gu, Daejeon, 35365 Republic of Korea

**Keywords:** Cricoid compression, Esophagus, Pediatric patients, Ultrasonography

## Abstract

**Background:**

The use of cricoid compression to prevent insufflation remains controversial, and its use in children is limited. This study aimed to examine the effect of real-time ultrasound-guided esophageal compression on the prevention of gastric insufflation.

**Method:**

This prospective observational study was conducted with fifty children aged < 2 years undergoing general anesthesia. Patients were excluded if they were at an increased risk for gastric regurgitation or pulmonary aspiration. Following anesthetic induction under spontaneous breathing, ultrasound-guided esophageal compression was performed during pressure-controlled face-mask ventilation using a gradual increase in peak inspiratory pressure from 10 to 24 cm H_2_O to determine the pressure at which gastric insufflation occurred. The primary outcome was the incidence of gastric insufflation during anesthetic induction with variable peak inspiratory pressure after real-time ultrasound-guided esophageal compression was applied.

**Results:**

Data from a total of 42 patients were analyzed. Gastric insufflation was observed in 2 (4.7%) patients. All patients except one had their esophagus on the left side of the trachea. Applying ultrasound-guided esophageal compression did not affect the percentage of glottic opening scores (*P* = 0.220).

**Conclusions:**

The use of real-time ultrasound-guided esophageal compression pressure can aid preventing gastric insufflation during face-mask ventilation in children less than 2 years old.

**Trial registration:**

Clinicaltrials.gov identifier: NCT04645043.

**Supplementary Information:**

The online version contains supplementary material available at 10.1186/s12871-022-01803-5.

## Background

Face-mask ventilation is a method of supplying oxygen before achieving a confirmed and secured airway by intubation during anesthetic induction. However, this method can cause gastric insufflation through the esophagus along with ventilation through the airway. Furthermore, ventilation difficulty can result from an increase in extrapleural pressure following gastric distension due to gastric insufflation [1–3].

Cricoid pressure is one of the methods that help prevent this adverse reaction through the compression of the esophagus with pressure on the cricoid cartilage, which prevents aspiration and gastric regurgitation [4, 5]. Cricoid pressure has been widely used for several decades; however, its usefulness has recently become controversial [4, 5]. Specifically, it may disturb ventilation and intubation in children [5]. Moreover, retrospective evaluation of computed tomography (CT) images in children revealed that lateral deviation of the esophagus to the airway occurred in 45% of younger children (< 8 years), and differed significantly to that observed in older children [6]. CT and magnetic resonance imaging (MRI) of awake volunteers revealed that the esophagus exhibited lateral deviation in half of the volunteers, and cricoid pressure caused displacement of the esophagus in 90.5% of them [7]. In particular, the application of cricoid pressure in patients less than 2 years old,where airway management may be relatively difficult, may lead to unexpected situations [8].

With real time ultrasound, we can easily check the location of esophagus and compressed esophageal lumen. Several case reports regarding the use of this method in adults have been conducted previously [9, 10]. Therefore, we hypothesized that real-time ultrasound-guided esophageal compression would reduce the risk of gastric air insufflation without making mask ventilation or laryngoscopy more difficult in infants and children.

## Methods

### Study design and settings

The Institutional Review Board of Seoul National University Hospital approved this study (approval no. 2009–150-1160, approval date 17/11/2020, chairperson prof. Park byung-joo). This study was registered at clinicaltrials.gov (NCT04645043, first posted date: 25/11/2020). The first participant was enrolled on 30/11/2020 and the last participant was followed up on 18/03/2021. This was conducted in accordance with the tenets of the Declaration of Helsinki. This prospective observational study was conducted at Seoul National University Children’s Hospital.

### Participants

This study included patients aged < 2 years who underwent general anesthesia. Patients were excluded if they were at an increased risk for regurgitation or pulmonary aspiration due to factors such as hypertrophic pyloric stenosis, delayed gastric emptying time, or esophageal stricture; difficult visualization of the stomach via ultrasound was expected due to factors such as paralytic ileus, bowel perforation, hepatomegaly, splenomegaly; or there were air artefacts in the stomach at baseline gastric ultrasound examination. One investigator explained the study protocol the day before the surgery and obtained informed consent from the guardians of the pediatric patients.

### Variables

The primary outcome was the incidence of gastric insufflation during positive pressure ventilation by mask after induction of anesthesia. Varying peak inspiratory pressure (increasing from 10 cm H_2_O to 24 cm H_2_O) were used while ultrasound guided esophageal compression was performed. Gastric insufflation was detected by real time ultrasound examination of the antrum (acoustic shadow phenomenon and/or a comet-tail artifacts) [11]. The secondary outcomes included 1) estimation of minimal inspiratory pressure at which gastric insufflation occurs; 2) position of the esophagus relative to the trachea; 3) tracheal insufflation after applying ultrasound-guided esophageal pressure; 4) changes in the percentage of the glottis opening (POGO) score after applying ultrasound-guided esophageal was applied.

### Conduct of the study and data measurements

#### Anesthesia and positive pressure ventilation

**Fig. 1:**
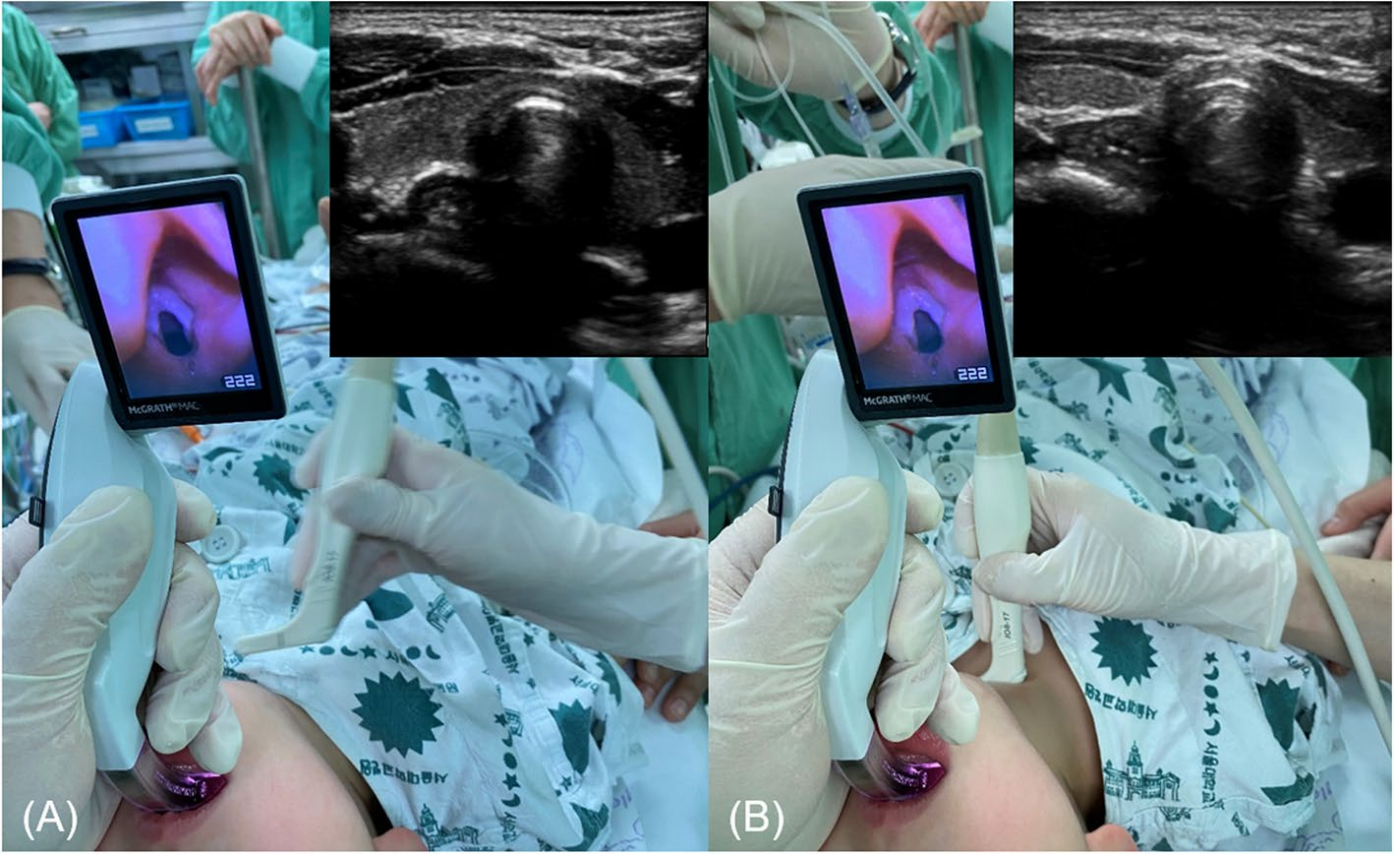
Glottic opening score percentages before (**A**) and after (**B**) ultrasound-guided esophageal compression was applied in a boy aged 23 months who weighed 12.5 kg. Before (**A**) and after (**B**) ultrasound-guided esophageal pressure application in a girl aged 14 months and weighing 12.2 kg

Anesthesia was induced with atropine (0.02 mg.kg^−1^) and thiopental (5 mg.kg^−1^) along with oxygen through a face mask while the patient was monitored using electrocardiography, pulse oxygen saturation, and non-invasive blood pressure monitoring. Sevoflurane (3–5 vol%) was administered via spontaneous ventilation. After loss of consciousness, the baseline gastric ultrasound examination was conducted and the gastric antral area (GAA) was measured with the linear-shaped L3-12 T ultrasound transducer, E-CUBE i7 (ALPINION Medical Systems Co., Seoul, Korea) using the following formula: GAA = π × D1 × D2 × 4^–1^ [11]. Subsequently, a neuromuscular blocking agent (rocuronium, 0.6 mg.kg^−1^) was administered, and positive pressure ventilation was started with a two-handed technique and jaw thrust on the tight sealing of the face mask with 100% oxygen flow at 2 l.min^−1^. Ventilation was controlled using a pressure-controlled mode, which maintained the esophageal pressure. The first inspiratory pressure was 10 cm H_2_O, which was sequentially increased to a maximum of 24 cm H_2_O. At least three ventilations were applied to the patient at each inspiratory pressure level. If any evidence of gastric insufflation during the increase in inspiratory pressure was observed, the investigation was halted and the pressure that achieved gastric insufflation and the GAA just before that time was recorded. If no gastric insufflation was observed at any inspiratory pressure, the investigation was stopped, and the GAA at the maximum inspiratory pressure was recorded. The study was also stopped if the peak inspiratory pressure applied resulted in a tidal volume was > 20 ml.kg^−1^. After finishing the investigation via an increase in the inspiratory pressure, the POGO score was recorded using the videolaryngoscope (McGRATH™ MAC Video Laryngoscope) under esophageal compression, and intubation proceeded (Fig. 1). Gastric air was suctioned after finishing the measurements to decompress the stomach if gastric insufflation was observed during the measurement.

#### Ultrasound assessments of esophagus and stomach

**Fig. 2:**
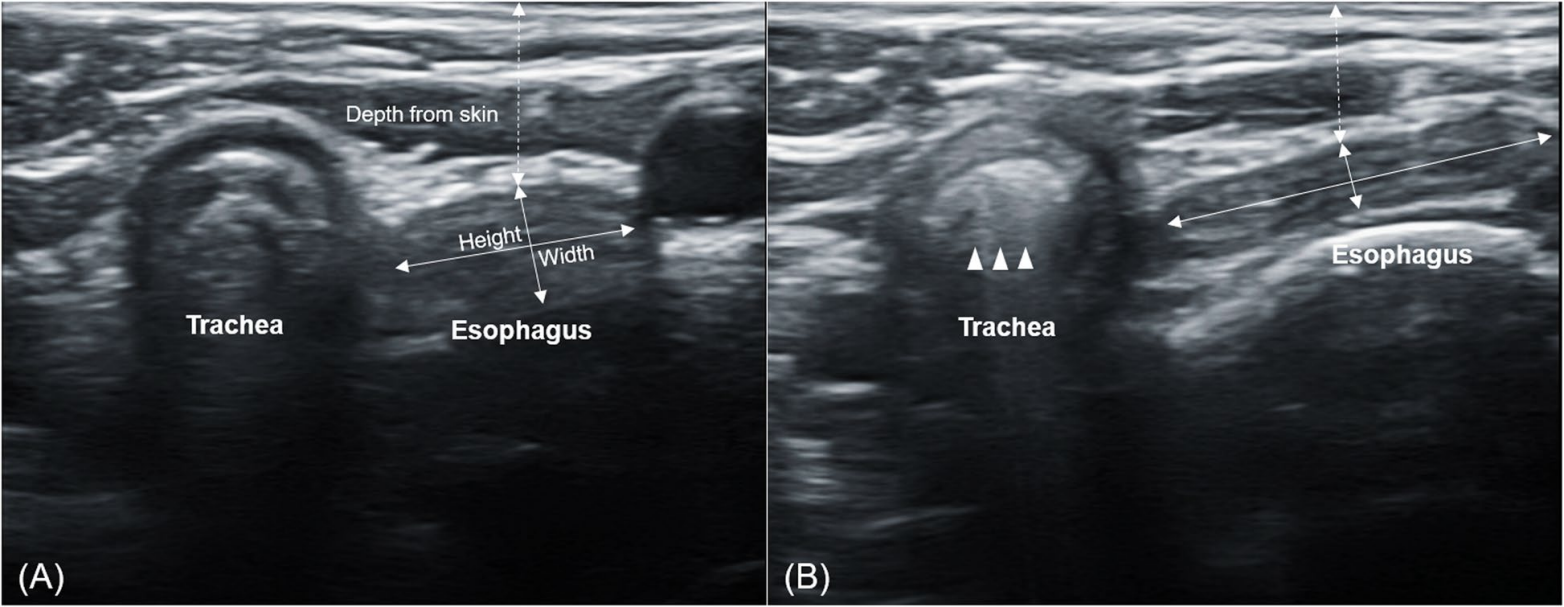
Before (**A**) and after (**B**) ultrasound-guided esophageal pressure application in a girl aged 14 months and weighing 12.2 kg. The arrows indicate tracheal insufflation during positive pressure ventilation

All investigations in the present study were conducted in the supine position. One investigator checked the location of esophagus and compressed the esophagus at the cricoid level for collapsing the esophageal lumen using ultrasound Sonosite X-porte (FUJIFILM SonoSite, Inc. Bothell, WA, USA) with an HSL25xp 13–6 MHz linear probe. The investigator measured the depth from the skin to the esophagus and the esophageal width and height before and after applying ultrasound-guided compression. The presence of esophageal and tracheal air insufflation was monitored during positive pressure ventilation (Fig. 2). When an acoustic shadow phenomenon and/or a comet-tail artifact in the esophagus appeared on the ultrasound, indicating esophageal insufflation, the pressure at that time was recorded. We checked whether tracheal insufflation was present to evaluate proper oxygenation when applying ultrasound-guided esophageal compression.

Another investigator monitored the occurrence of gastric insufflation concurrently at the level of the gastric antrum with gastric ultrasonography, using a linear-shaped L3-12 T transducer, E-CUBE i7 (ALPINION Medical Systems Co., Seoul, Korea), during positive pressure ventilation. The gastric antrum was identified between the left lobe of the liver and the pancreas in the sagittal plane to the right parasagittal plane at the level between the aorta and the superior mesenteric artery or the inferior vena cava, after which the transducer was tilted and rotated to the long axis of the antrum [11].

### Sample size

To our knowledge, no previous studies have evaluated the effect of ultrasound-guided esophageal compression on the incidence of gastric insufflation. Considering the exploratory nature of this study, we assumed that 50 pediatric patients were sufficient to demonstrate the effect of ultrasound-guided esophageal compression.

### Statistical analysis

All continuous variables are presented as mean ± standard deviation (SD) or median with interquartile range (IQR), depending on whether the variable was parametric. The normal distribution assumption was verified using the Kolmogorov–Smirnov test. The esophageal depth from the skin and esophageal width, height, and POGO score before and after ultrasound-guided esophageal compression application were assessed using a paired t-test. Statistical analyses were performed using SPSS version 18 software (SPSS Inc., Chicago, IL, USA). Differences were considered statistically significant at *P* < 0.05.

## Results

Fifty pediatric patients were enrolled in this study. After eight patients showing gastric insufflation in the baseline examination were excluded, data from 42 patients were included in the final analysis.

**Fig. 3:**
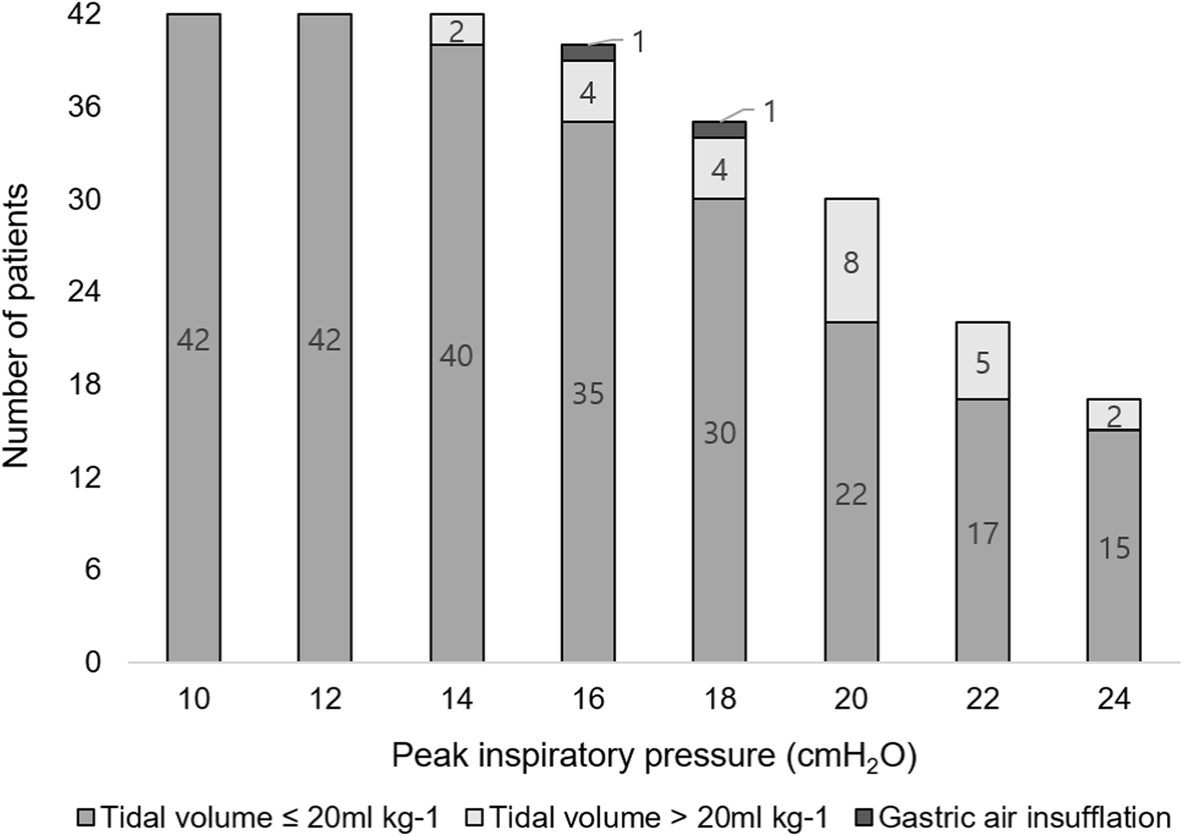
Number of patients at each peak inspiratory pressure

The patients’ characteristics are presented in Table 1. Figure 3 presents the number of patients at each peak inspiratory pressure. Gastric insufflation occurred in 2 (4.7%) patients. The others did not show gastric insufflation. In these patients, the maximal peak inspiratory pressure was 20.8 (3.2) cm H_2_O.

**Table 1:** Patients’ chractertistics

	*N* = 42
Age (months)	11. 6 ± 6.5
Height (cm)	73.7 ± 10.6
Weight (kg)	9.6 ± 2.5
Gender (male, %)	31, 73%

Data presented as mean ± SD or number

Table 2 presents ultrasound assessment data obtained during ultrasound-guided esophageal compression application. The peak inspiratory pressure values for gastric insufflation were 16 and 18 cm H_2_O in two patients who had gastric insufflation despite of ultrasound-guided esophageal compression, respectively. Esophageal ultrasound detected esophageal insufflation in one of these patients. Esophageal air insufflation was observed in four patients at peak inspiratory pressures of 14, 16, 16, and 22 cm H_2_O, respectively. Among them, three did not experience gastric insufflation despite the occurrence of esophageal insufflation.

**Table 2:** Ultrasound assessment during the ultrasound guided esophageal pressure application

		*N* = 42	
Gastric air insufflation (n, %)		2, 4.7	
Esophageal air insufflation (n, %)		4, 9.5	
Esophagus position (Left side to trachea, %)		41, 97.6	
Tracheal air insufflation (n, %)		42, 100	
Maximal peak inspiratory pressure (cmH_2_0)		20.8 ± 3.2	
Gastric antrum CSA (cm^2^)		1.0 ± 0.3	
	**Baseline**	**Ultrasound guided esophageal pressure applied**	***P* value**
Esophagus depth from skin (mm)	9.8 ± 2.3	7.2 ± 1.9	< 0.001
Esophagus width (mm)	6.5 ± 1.5	7.3 [6.4–8.4]	0.020
Esophagus height (mm)	4.4 [3.9–5.3]	3.6 [2.8–4.0]	< 0.001
POGO score	100 [100–100]	100 [100–100]	0.220

Data presented as mean ± SD, number or median (IQR). *CSA* cross sectional area, *POGO* percentage of glottis opening score

The esophagus of every patient except one was located left to the trachea, while that of the other was located posterior to the trachea. The POGO score did not differ before and after ultrasound-guided esophageal compression application (*P* = 0.220; 95% CI, -1.33–5.62).

## Discussion

This study is the first prospective study to confirm that real-time ultrasound-guided esophageal compression can be safely applied to prevent gastric insufflation in infants and children. The location of the esophagus relative to the trachea was also laterally displaced in 97.6% of the patients in the present study.

Cricoid pressure, first found in the literature in 1774 and contributed by Sellick in 1961, may help to prevent gastric insufflation during positive ventilation; however, controversy regarding its use remains. In particular, routine use of this method on patients aged < 1 years by pediatric anesthesiologists occurs in less than half of their practices, and the risk of trauma associated with the compression of the surrounding structures and trachea is still a concern [4, 12]. In addition, pressing with a force of 30 N is required to achieve cricoid pressure in adults; meanwhile, a force of 7.7 N is sufficient for children [5]. Nevertheless, this method is potentially dangerous and determining how much pressure is exerted when performed blindly in the absence of feedback is challenging. In this study, we were able to proceed safely, using ultrasound to monitor the pressure on half of the esophageal structure.

As a result, the incidence of gastric insufflation in the present study was 4.7%, suggesting that this approach may be effective as compared to various studies confirming gastric insufflation [3, 13–15]. The incidence of gastric insufflation during face-mask ventilation in adults and children in previous studies was in the range of 58–73% [3, 13–15]; in one study, it was 17% despite the application of cricoid pressure [13]. In addition, the minimal peak inspiratory pressure that increases the risk of gastric insufflation was 20.8 cm H_2_O, which was higher than the 12 and 18 cm H_2_O observed during previous studies on children [3, 15], or the 14 and 20 cm H_2_O reported in adults with and without the use of cricoid pressure, respectively [13].

Meanwhile, tracheal insufflation was confirmed in all patients in the present study (Additional File 1), and the POGO score did not differ before and after ultrasound-guided esophageal compression application. Difficulty of mask ventilation and laryngoscopic view one reason for clinicians’ reluctance to apply cricoid pressure in children. In addition, using conventional cricoid pressure may worsen the laryngoscopic view without sufficient benefits to justify routine use [5]. However, real-time ultrasound-guided esophageal compression application can effectively prevent gastric insufflation without causing ventilation difficulty or changes in the laryngoscopic view, which is more practical, despite concerns that applying cricoid pressure during intubation makes intubation difficult.

The posterior tracheal ring is expected to compress the esophagus by compressing the trachea at the cricoid cartilage level during the application of conventional cricoid pressure [16]. However, conventional cricoid pressure cannot effectively compress the esophagus [17], which is located on the left side in the patients included in the present study. Previous studies have demonstrated that the esophagus may be displaced at the cricoid cartilage level when cricoid pressure is applied, as confirmed in both CT and MRI in children and adults. Lateral displacement of the esophagus was observed in all patients (97.6%) included in the present study, except one. Applying cricoid pressure may also result in esophageal injury or fractures in the cricoid cartilage [5], however, the use of ultrasound-guided esophageal pressure may help prevent these side effects. Therefore, if pressure is applied to the esophagus with the real-time ultrasound-guided esophageal compression method, the risk of trauma may be reduced due to the on-going monitoring of surrounding structures, and the accompanying reduction in esophageal insufflation and gastric insufflation risk.

This study has some limitations. First, this study was conducted as an observational study to determine the usefulness of real-time ultrasound-guided esophageal pressure and sample size could not be estimated before conducting this study; therefore, biases related to observational studies, such as confounders and selection bias, could not be assessed. However, the results obtained in this study may be comparable with other studies that did not use ultrasound-guided esophageal compression even though this study was not performed as a randomized controlled trial. Second, we excluded patients with hypertrophic pyloric stenosis or delayed gastric emptying time, both of which increase the risk of aspiration; therefore, further research regarding this matter is needed. Also, gastric insufflation may be affected by obtrsuctive airway states. Gastric insufflation in this study was confirmed in a state with no observed airway obstruction, and there is a limit to applying it in the presence of airway obstruction. Third, a neuromuscular blocking agent was used during this study; however, positive ventilation was performed in the absence of self-ventilation instead of a train of four monitors, possibly affecting insufflation. Lastly, even though measurements performed in the left lateral decubitus position via gastric ultrasonography are more accurate for detecting gastric insufflation, the supine position was used in the present study, which may have affected the findings. Therefore, we may not have detected the small quantities of air that would not have reached the antrum in the supine position.

## Conclusions

In conclusion, applying real-time ultrasound-guided esophageal compression can help prevent gastric insufflation without causing ventilation difficulty during mask ventilation in small children.

## Supplementary Information

**Additional file 1.** 

## Data Availability

The datasets generated and/or analysed during the current study are not publicly available, but are available from the corresponding author on reasonable request.

## Abbreviations

CT: Computed tomography
MRI: Magnetic resonance imaging
POGO: The percentage of the glottic opening
GAA: Gastric antral area
SD: Standard deviation
IQR: Interquartile range
