# Supplementary material for: Ultrasound-guided esophageal compression during mask ventilation in small children: a prospective observational study
Source: BMC Anesthesiol. 2022 Aug 15;22:257. doi: 10.1186/s12871-022-01803-5 (PMC9377106; doi:10.1186/s12871-022-01803-5)
Supplement: Supplementary file 1 — Additional file 1. [file 12871_2022_1803_MOESM1_ESM.docx]

**Supplemental digital content:**

Supplemental Digital Content1.wmv

The supplemental video clip shows esophageal and tracheal air insufflation before and after ultrasound-guided esophageal compression application. Ultrasound-guided esophageal compression prevents esophageal air insufflation at a peak inspiratory pressure of 20 cm H2O, maintaining tracheal air insufflation. The written consent from the legal guardians of the patient was obtained.
